# The monetary burden of cysticercosis in Mexico

**DOI:** 10.1371/journal.pntd.0007501

**Published:** 2019-07-10

**Authors:** Rachana Bhattarai, Hélène Carabin, Jefferson V. Proaño, Jose Flores-Rivera, Teresa Corona, Ana Flisser, Leith León-Maldonado, Christine M. Budke

**Affiliations:** 1 Department of Veterinary Integrative Biosciences, College of Veterinary Medicine & Biomedical Sciences, Texas A&M University, Texas, United States of America; 2 Département de Pathologie et Microbiologie, Faculté de Médecine Vétérinaire, Université de Montréal, Saint-Hyacinthe, Canada; 3 Hospital de Especialidades, Centro Médico Nacional Siglo XXI, Instituto Mexicano del Seguro Social, Mexico City, Mexico; 4 Clinical Laboratory of Neurodegenerative Diseases, National Institute of Neurology and Neurosurgery, Mexico City, Mexico; 5 Facultad de Medicina, Universidad Nacional Autónoma de México, Mexico City, Mexico; 6 Cátedra CONACYT, Instituto Nacional de Salud Pública, Cuernavaca, Mexico; IRNASA, CSIC, SPAIN

## Abstract

**Background:**

*Taenia solium* cysticercosis is a public health and agricultural problem in many low and middle-income countries where health education, sanitation, pig management practices and meat inspection infrastructure are insufficient. Cysticercosis affects both human and animal health and has important economic consequences. Very few studies have been conducted to evaluate the monetary burden of cysticercosis. This study aimed at estimating the 2015 costs associated with cysticercosis in humans and pigs in Mexico.

**Methods:**

The monetary burden of human cysticercosis was estimated based on costs incurred by living with and treating epilepsy and severe chronic headaches associated with neurocysticercosis (NCC). The estimated cost of porcine cysticercosis took into consideration losses due to the reduction in the price of cysticercosis-infected animals. Epidemiologic and economic data were obtained from the published literature, government reports, and setting-specific questionnaires. Latin hypercube sampling methods were employed to sample the distributions of uncertain parameters and to estimate 95% credible regions (95% CRs). All results are reported in 2015 U.S.$.

**Findings:**

The overall monetary burden associated with NCC morbidity was estimated at U.S.$215,775,056 (95% CR U.S.$109,309,560 –U.S.$361,924,224), with U.S.$436 (95% CR: U.S.$296 –U.S.$604) lost per patient. If loss of future years of income and productivity due to NCC-associated deaths was included, this value increased by U.S.$54.26 million, assuming that these individuals earned Mexico’s median wage salary. An additional U.S.$19,507,171 (95% CR U.S.$5,734,782 –U.S.$35,913,487) was estimated to be lost due to porcine cysticercosis.

**Conclusions:**

This study suggests that *T*. *solium* cysticercosis results in considerable monetary losses to Mexico.

## Author summary

*Taenia solium* cysticercosis is a parasitic disease, which can be transmitted between humans and pigs in areas of the world where pigs are raised in a traditional manner and latrines are not always used by the local population. Because cysticercosis affects humans and pigs, it is important to assess how much this infection costs both the public health and agricultural sectors in endemic countries such as Mexico. Costs associated with infection of both humans and pigs, in Mexico, were estimated using the literature, questionnaires, and publicly available data. Study findings suggest that *T*. *solium* cysticercosis results in considerable monetary losses to Mexico. The methodology developed here can be applied to estimate the monetary burden of cysticercosis in other regions in order to better prioritize disease control initiatives.

## Introduction

Cysticercosis is a public health and agricultural problem caused by the larvae of the zoonotic cestode *Taenia solium*. Humans are the definitive hosts of *T*. *solium*, with adult tapeworms found in the intestines after ingestion of undercooked pork containing cysticerci. Infection with the adult stage of the parasite is known as taeniasis. Pigs acquire cysticercosis when ingesting eggs shed in the feces of humans with taeniasis. Porcine cysticercosis results in the development of cysts in the muscles, including the tongue, heart and diaphragm, the brain, and other organ systems [1]. When humans accidentally ingest eggs shed in the feces of an infected human, they develop larval cysts (cysticercosis) similar to infected pigs. Neurocysticercosis (NCC) occurs when *T*. *solium* cysticerci infect the central nervous system, which can result in symptoms such as epilepsy, severe chronic headaches, hydrocephalus, stroke, and dementia [2].

Porcine cysticercosis and NCC have important economic consequences [3,4]. NCC incurs direct and indirect costs. Direct costs include fees associated with medical services, diagnostic procedures, surgical interventions, prescribed chemotherapeutic treatment, hospitalization, and traditional therapies. Indirect costs are associated with loss of working days due to visits to a healthcare provider or hospitalization, loss of working days due to illness not requiring immediate medical attention, over-the-counter medication, loss of income due to reduced productivity, transportation to and from medical treatment, and time lost by the patient’s family to take care of them or to accompany them to receive medical care [5] [6]. In pigs, cysticercosis can lead to partial or full condemnation of the carcass and economic losses in areas where meat is inspected [7] [8]. In some areas, pigs are evaluated for the presence of cysts in the tongue via palpation. The owner of a pig suspected of being infected with cysticercosis will usually receive a lower price for the animal or will be unable to sell the animal. NCC has been shown to result in a significant economic burden to people in Mexico requiring hospitalization [9]. However, no previous study has evaluated the burden of cysticercosis in Mexico incorporating both human and pig losses. Cysticercosis-associated monetary losses to both the human health and agricultural sectors have been evaluated in South Africa, Lao People's Democratic Republic, Cameroon, Tanzania, and India [3,4,10–12]. Studies are needed to estimate the burden of cysticercosis in endemic countries to facilitate comparisons with other locally important health conditions and to better prioritize disease control initiatives. The research presented here provides the first estimate of the monetary burden of human NCC-associated epilepsy and severe chronic headaches and porcine cysticercosis for the country of Mexico.

## Materials and methods

### Study area

Mexico is the third largest country in Latin America, with a 2015 population of almost 125 million and an annual population growth rate of 1.18% [13]. It is the eleventh most populous country in the world, with 21% of the population living in rural areas [13]. Traditional pig rearing practices in *T*. *solium*-endemic areas allow pigs to have access to human feces in open fields facilitating the completion of the *T*. *solium* life cycle [14,15]. Confined pigs in yards next to dwellings may also have direct access to poorly maintained outdoor latrines [16].

### Estimation of the number of NCC cases with epilepsy and severe chronic headaches in Mexico

The exact number of NCC cases in Mexico is not known. The proportion of people with NCC who develop epilepsy, severe chronic headaches or other clinical manifestations is also unknown. Therefore, the numbers of cases of NCC-associated epilepsy and NCC-associated severe chronic headaches in 2015, in urban and rural areas of Mexico, were estimated based on the model used in [17]. Details on the epidemiologic parameters used to estimate the number of NCC cases with epilepsy and severe chronic headaches are provided as supporting information (S1 Supporting Information).

### Treatment-seeking behavior of people with NCC in Mexico

**Fig 1:**
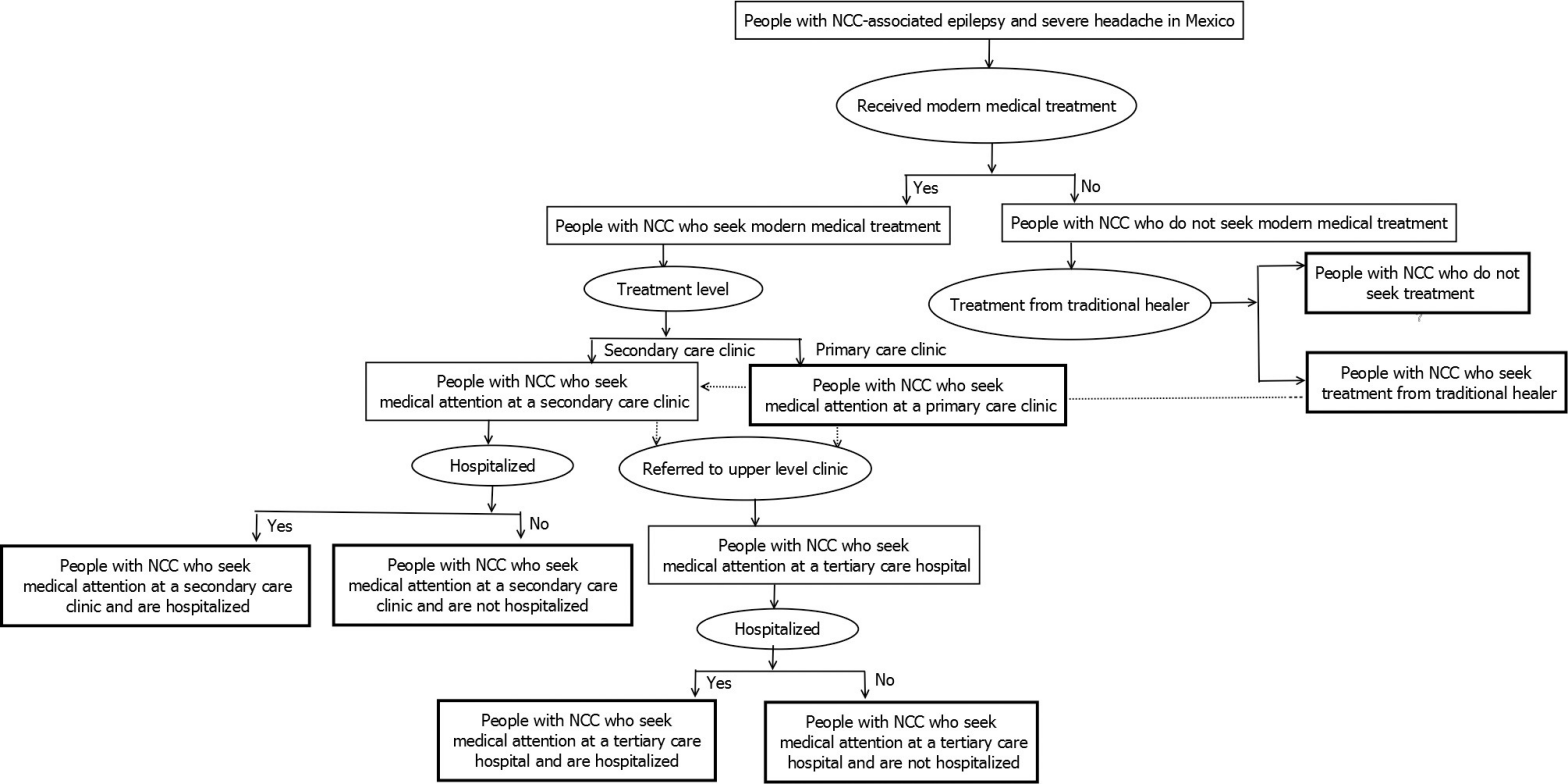
Flowchart showing categories of treatment-seeking behavior of people with NCC-associated epilepsy and NCC-associated severe chronic headaches in Mexico. Note: Please refer to Table 1 for information concerning the uncertainty distributions associated with the specific parameters. All data were stratified by rural/urban residence. Localities of 2,500 or more inhabitants were considered urban (UN 2010).

People with NCC-associated epilepsy and severe chronic headaches were divided into two categories; 1) those who do not seek modern medical treatment, and 2) those who seek modern medical treatment. Modern medical treatment is defined as western medicine. These categories were further divided into sub-categories (see the next sections). Fig 1 depicts the treatment end-points for Mexicans with NCC-associated epilepsy and severe chronic headaches. Literature-based information on healthcare-seeking behavior and treatment gaps was used to estimate the number of people with NCC in each of the above groups. A setting-specific questionnaire was developed in Spanish in 2014 to obtain information not found in the published literature. The details of this setting-specific questionnaire are presented as supporting information (S2 Supporting Information).

### People with NCC-associated epilepsy or severe chronic headaches seeking modern medical treatment

A recent systematic review of epilepsy and NCC in Latin America was used to estimate the number of NCC-associated epilepsy cases receiving modern medical treatment in urban and rural areas of Mexico [18]. The percentage of patients with severe chronic headaches not seeking modern medical care was assumed to be 10% more than that of epilepsy patients due to the generally greater clinical severity of epilepsy. This estimate was based on studies conducted in the United Kingdom (U.K.) where the epilepsy treatment gap was reported at 2%, while the migraine treatment gap was 14% [19,20] (Table 1). People with NCC receiving modern medical treatment were further broken down into six sub-categories representing the highest level of care obtained; i) those who receive medical attention from a primary care provider, ii) those who receive medical attention from both a primary care provider and a traditional healer, iii) those who receive medical attention from a secondary care provider and are not hospitalized, iv) those who receive medical attention from a secondary care provider and are hospitalized, v) those who receive medical attention at a tertiary care hospital and are not hospitalized, and vi) those who receive medical attention at a tertiary care hospital and are hospitalized.

**Table 1:** Human epidemiologic parameters used to estimate the monetary burden of cysticercosis in Mexico.

Parameter	Symptom	Area	Value	Distribution	Reference
Proportion not receiving modern medical treatment	Epilepsy	Urban	Min: 0.10 Max: 0.46	Uniform	[18]
		Rural	Min: 0.67 Max: 0.87	Uniform	[18]
	Severe chronic headaches	Urban	Min: 0.21 Max: 0.56	Uniform	[see text]
		Rural	Min: 0.78 Max: 0.97	Uniform	[see text]
Proportion exclusively seeking care from a traditional healer	Epilepsy	Urban	Min: 0 Mode: 0 Max: 0.35	Triangular	S2 Supporting Information
		Rural	Min: 0 Mode: 0 Max: 0.7	Triangular	S2 Supporting Information
	Severe chronic headaches	Urban	Min: 0 Mode: 0 Max: 0.4	Triangular	S2 Supporting Information
		Rural	Min: 0 Mode: 0 Max: 0.8	Triangular	S2 Supporting Information
Proportion seeking medical attention at a primary care clinic and referred to upper level care	Epilepsy	Urban	Min: 0.21 Max: 0.59	Uniform	[21,22]
		Rural	Min: 0.10 Max: 0.30	Uniform	[see text]
	Severe chronic headaches	Urban	Min: 0.08 Max: 0.30	Uniform	[23,24]
		Rural	Min: 0.04 Max: 0.15	Uniform	[see text]
Proportion referred to a tertiary hospital from a primary care clinic	Epilepsy	Urban	Min: 0 Mode: 0 Max: 0.8	Triangular	S2 Supporting Information
		Rural	Min: 0 Mode: 0 Max: 0.4	Triangular	S2 Supporting Information
	Severe chronic headaches	Urban	Min: 0 Mode: 0 Max: 0.2	Triangular	S2 Supporting Information
		Rural	Min: 0 Mode: 0 Max: 0.1	Triangular	S2 Supporting Information
Proportion referred to a tertiary care hospital from a secondary care clinic	Epilepsy	Urban	Min: 0 Mode: 0 Max: 0.5	Triangular	S2 Supporting Information
		Rural	Min: 0 Mode: 0 Max: 0.5	Triangular	S2 Supporting Information
	Severe chronic headaches	Urban	Min: 0 Mode: 0 Max: 0.25	Triangular	S2 Supporting Information
		Rural	Min: 0 Mode: 0 Max: 0.25	Triangular	S2 Supporting Information
Proportion of people seeking medical attention from a modern doctor and also receiving treatment from a traditional healer	Epilepsy	Urban	Min: 0.005 Mode: 0.025 Max: 0.5	Triangular	[see text]
		Rural	Min: 0.01 Mode: 0.05 Max: 1	Triangular	S2 Supporting Information
	Severe chronic headaches	Urban	Min: 0 Mode: 0 Max: 0.475	Triangular	[see text]
		Rural	Min: 0 Mode: 0 Max: 0.95	Triangular	S2 Supporting Information

In Mexico, people with epilepsy or severe chronic headaches generally initially seek treatment at a primary care clinic. Hence, all NCC patients seeking modern care were assumed to have visited a primary care physician. A proportion of NCC patients seen at a primary care clinic are referred for further treatment at a higher care facility. For epilepsy patients in urban areas, data from a 2007 study conducted in Brazil and a 2010 study conducted in the U.K. were used due to a lack of published data from Mexico. The proportion was modeled as a uniform distribution between the U.K. study (23%) and the Brazilian study (59%) [21,22]. Similarly, the proportion of people with severe chronic headaches referred to upper level care in urban areas was assumed to follow a uniform distribution between an estimate from a study of people with migraine conducted in Latin America in 2005 (8%) and a study conducted in the United States (U.S.) in 1993 (30%) [23,24]. A 2003 U.S. study showed that urban physicians are twice as likely to refer people for upper level care on the suspicion of hereditary breast cancer compared to rural physicians [25]. Since referral data based on a rural versus urban setting are not available from Mexico, the proportions of cases of epilepsy and severe chronic headaches referred in urban areas were multiplied by 0.5 to obtain the proportion of cases in rural areas.

The proportion of patients referred to a tertiary care hospital directly from a rural primary care clinic was based on questionnaire responses provided by physicians working in a primary care clinic in the state of Michoacán. The state of Michoacán was chosen since it is a known endemic area for cysticercosis and the authors had contacts within the local healthcare system and Ministry of Health that facilitated questionnaire distribution. The proportion of patients referred to a tertiary care hospital directly from a secondary care clinic was based on questionnaire responses provided by neurologists working in a secondary care clinic in Michoacán. The estimates were modeled as triangular distributions using the provided values for minimum, mode, and maximum. It was assumed that secondary care providers and tertiary hospitals are primarily located in urban areas; therefore, the proportions of patients that were referred to tertiary hospitals from secondary care clinics would be the same for both urban and rural areas.

The proportions of people with epilepsy or severe chronic headaches referred from a primary care provider to a secondary care provider, in rural areas, were estimated by subtracting the proportion of people referred to a tertiary care hospital from all referred patients with epilepsy and severe chronic headaches. Based on the assumption that the referral rate is double in urban areas, the values in rural areas were multiplied by two to estimate the proportions referred from primary to secondary care in urban areas.

### People with NCC-associated epilepsy or severe chronic headaches not seeking modern medical treatment

It was assumed that some individuals with epilepsy or severe chronic headaches seek treatment exclusively from traditional healers. Questionnaire findings from employees at the Michoacán Office of the Ministry of Health estimated that an average of 23% (min: 0%, mode: 0%, max: 70%) and 26% (min: 0%, mode: 0%, max: 80%) of people with epilepsy and severe chronic headaches from rural areas, respectively, seek medical attention exclusively from traditional healers. Questionnaire findings are in line with a 2015 study conducted in rural Mexico where 33% of people with epilepsy sought treatment from traditional healers after their first seizure [26]. There was no literature to support the proportion of people with severe chronic headaches seeking treatment from traditional healers in Mexico. However, questionnaire findings are similar to those of a study conducted in rural and urban Taiwan where 24% of people with migraines sought treatment from practitioners of traditional Chinese medicine [27]. Triangular distributions using the minimum, mode, and maximum values from the questionnaires were used to estimate the number of NCC-associated epilepsy and severe chronic headaches cases seeking treatment solely from traditional healers in rural areas of Mexico.

According to Hoeven et al. 2012, individuals residing in rural areas of South Africa were twice as likely to prefer treatment from a traditional healer compared to individuals residing in urban areas [28]. Since such data are not available from Mexico, the proportions of individuals with epilepsy and severe chronic headaches who seek medical attention exclusively from a traditional healer in rural areas were multiplied by 0.5 to obtain the proportions of individuals who seek medical attention exclusively from a traditional healer in urban areas. The numbers of NCC-associated epilepsy and severe chronic headaches cases seeking treatment solely from traditional healers were estimated by multiplying the number of NCC-associated epilepsy and NCC-associated severe chronic headaches cases in rural and urban areas by the proportion of people seeking treatment from traditional healers (Table 1).

The proportion of people with NCC-associated epilepsy or severe chronic headaches who do not receive any treatment was estimated by subtracting the proportion of people who only receive treatment from traditional healers from the proportion of people who do not seek modern medical treatment. These proportions were multiplied by the numbers of people with NCC-associated epilepsy and severe chronic headaches in rural and urban areas to obtain the respective numbers of people with NCC not receiving any form of treatment.

### People with NCC-associated epilepsy or severe chronic headaches seeking both traditional and modern medical treatments

Questionnaire responses provided by employees of the Michoacán branch of Mexico’s Ministry of Health were used to estimate the proportion of NCC patients seeking care from both modern and traditional medicine in rural areas (35% for epilepsy and 31% for severe chronic headaches; see S2 Supporting Information). The estimate provided is consistent with findings from a study conducted in the Rio Grande Valley of Texas where 44% of Mexican Americans were found to use alternative medicine in addition to modern medicine [29]. Based on the assumption that individuals residing in rural areas are twice as likely to prefer treatment from a traditional healer compared to individuals residing in urban areas, the above values were multiplied by 0.5 to estimate the proportion of people who seek medical attention from both a modern doctor and a traditional healer in urban areas.

### Parameters associated with use of healthcare resources among NCC patients in Mexico

Parameters associated with the use of healthcare resources, including diagnostic testing and prescribed medications, by people with NCC-associated epilepsy and severe chronic headaches are provided as supporting information (S3 Supporting Information). For patients seeking care from primary care clinics, the frequency of doctor visits and prescribed medications were based on data provided by primary care physicians in Michoacán. For patients referred to secondary care clinics, the frequency of doctor visits, medications, diagnostic tests such as computed tomography (CT) scans and magnetic resonance imaging (MRI), and hospitalization were based on data provided by neurologists working at a secondary care clinic in Michoacán. Data on frequency of electroencephalogram (EEG) and cerebral spinal fluid (CSF) testing for people who received treatment at a secondary care clinic were not available from study questionnaires and were assumed the same as for people seen in tertiary care facilities.

For people referred to a higher level of care, a single consultation with a healthcare provider was attributed to the referring lower level facility or facilities. For patients referred to tertiary health care settings, the frequency of doctor visits, medications, hospitalizations, surgical intervention, and diagnostic tests, including CT scans, MRIs, CSF testing, EEGs, enzyme-linked immunoelectrotransfer blots (EITBs), and enzyme-linked immunosorbent assays (ELISAs) were based on the results of a recent study conducted in two tertiary care hospitals in Mexico City, Mexico [9]. It was assumed that all NCC-related surgical interventions were performed at a tertiary care facility. Patients with epilepsy and severe chronic headaches seeking care in primary and secondary care facilities were assumed to receive the same non-NCC specific medical treatment protocols received by people with epilepsy and severe chronic headaches treated in tertiary care hospitals [9]. The assumption was also made that general drugs used to treat epilepsy and severe chronic headaches were available in both urban and rural settings. However, since diagnostic tests, including CT, MRI, and serology, are not typically available at primary care clinics, it was assumed that antihelminthic treatment was only prescribed in secondary and tertiary care clinics. Data on length of hospitalization in a secondary care facility were not available from the questionnaires and hospital stay length was assumed the same as that observed for non-surgical cases hospitalized at a tertiary care facility.

People with epilepsy exclusively seeking traditional medicine were assumed to visit a healer 4 to 6 times per year. These values were chosen in light of available data from a cross-sectional study conducted in 2002 in India suggesting that individuals with epilepsy visited a traditional healer 1 to 8 times per year [30]. For severe chronic headaches, the number of visits was assumed to be only 2 to 3 times per year, due to lesser clinical severity. It was also assumed that the number of visits to a traditional healer would be less for those people who seek medical attention from both a modern doctor and a traditional healer (2 to 3 times and 1 to 2 times per year for people with epilepsy and severe chronic headaches, respectively).

### Parameters associated with productivity losses in people with NCC

Table 2 shows the parameters associated with productivity losses in people with NCC. Information on loss of working days due to NCC-associated epilepsy and severe chronic headaches among patients seeking medical attention at a primary care clinic was based on values provided by physicians at a primary care clinic in Michoacán. Information for patients seeking medical attention at tertiary care hospitals was based on a study conducted in a referral hospital in Mexico [9]. People seeking care in secondary healthcare facilities were assumed to lose 25% fewer working days than those pursuing care at tertiary care facilities. For cases not seeking modern medical treatment, productivity losses were based on values provided by employees of the Michoacán branch of Mexico’s Ministry of Health.

**Table 2:** Parameters associated with productivity losses in people with NCC-associated epilepsy or severe chronic headaches in Mexico.

Parameter	Symptom	Hospitalized	Value	Distribution	Reference
Number of working days lost per year by people who seek medical attention at a primary care clinic	Epilepsy	No	Min: 0 Mode: 12 Max: 36	Triangular	S2 Supporting Information
	Severe chronic headaches	No	Min: 0 Mode: 12 Max: 24	Triangular	S2 Supporting Information
Number of working days lost per year by people who seek medical attention at a secondary care clinic	Epilepsy	No	18.75	Fixed	[see text]
		Yes	46.5	Fixed	[see text]
	Severe chronic headaches	No	12	Fixed	[see text]
		Yes	28.5	Fixed	[see text]
Number of working days lost per year by people who seek medical attention at a tertiary care hospital	Epilepsy	No	25	Fixed	[9]
		Yes	62	Fixed	[9]
	Severe chronic headaches	No	16	Fixed	[9]
		Yes	38	Fixed	[9]
Number of working days lost per year by people who do not seek treatment from a modern doctor	Epilepsy		Min: 12 Mode: 24 Max: 120	Triangular	S2 Supporting Information
	Severe chronic headaches		Min: 12 Mode: 12 Max: 60	Triangular	S2 Supporting Information
Proportion of Mexican adults that are not considered economically active excluding retirees	Epilepsy and severe chronic headaches		0.41	Fixed	[31]

### Epidemiologic parameters for porcine cysticercosis

Epidemiologic parameters for porcine cysticercosis are presented in Table 3. The number of pigs slaughtered in Mexico was obtained from the United States Department of Agriculture (USDA) Foreign Agricultural Service for the year 2015 [32]. Proportions of pigs slaughtered in federally inspected, municipal, and in-situ facilities were obtained for the year 2009 since this was the only year for which slaughter numbers were reported by facility type [33]. These proportions were then applied to the 2015 pig population. In-situ facilities are those without inspection, including home slaughtering. The prevalence of porcine cysticercosis was assumed lower in federally inspected and municipal facilities because most pigs slaughtered there would come from industrialized establishments. The prevalence of porcine cysticercosis at in-situ facilities was assumed to vary between 5% and 33% based on a study conducted in 13 villages located in the Sierra de Huautla region of Morelos, Mexico [14]. Due to limited data on porcine cysticercosis in pigs slaughtered in federally inspected and municipal facilities, the prevalence was assumed to be between 0 and 0.05%. This value seems reasonable when compared with a study conducted in Brazil from 2008 to 2013 where the prevalence of porcine cysticercosis in pigs reared under an intensive management system was 0.009% [34].

**Table 3:** Epidemiologic parameters used to estimate the monetary burden of porcine cysticercosis in Mexico.

Parameter	Facilities	Value	Distribution	Reference
Number of pigs slaughtered	Federally inspected, municipal, and in-situ facilities	17,315,000	Fixed	[32]
Proportion of pigs slaughtered	Federally inspected facilities	0.41	Fixed	[33]
	Municipal facilities	0.34	Fixed	[33]
	In-situ facilities	0.25	Fixed	[33]
Prevalence of porcine cysticercosis in pigs slaughtered	Federally inspected and municipal facilities	Min: 0 Max: 0.0005	Uniform	[see text]
	In-situ facilities	Min: 0.05 Max: 0.33	Uniform	[14]

### Human and pig economic parameters

Table 4 contains the economic parameters used to estimate the monetary burden of cysticercosis in Mexico in 2015 U.S. dollars. Direct costs included fees associated with medical services, diagnostic procedures, surgical interventions, prescribed chemotherapeutic treatment, hospitalization, and traditional therapies. The cost of doctor visits, diagnostic techniques and tests, a one-day stay in the hospital, and surgery were obtained from the 2006 standardized tariffs for healthcare services in Mexico [35]. Year 2006 tariffs were used due to their availability to study personnel and the period when data collection had occurred among patients seeking care in two tertiary hospitals in Mexico City [36]. The costs of medications used by people with NCC were based on year 2006 prices obtained from several pharmacies in Mexico. All 2006 costs were converted to the 2015 value according to the general Consumer Price Index for Mexico [37]. The cost for a visit to a traditional healer to treat epilepsy or severe chronic headaches was based on the minimum, mode, and maximum values provided by employees of the Michoacán branch of the Ministry of Health who completed the questionnaire.

**Table 4:** Economic parameters used to estimate the monetary burden of cysticercosis in Mexico (in 2015 U.S.$).

Parameter	Value	Distribution	Reference
Cost of a visit to a general practitioner/ neurologist/neurosurgeon	19	Fixed	[35]
Cost of a CT scan	194	Fixed	[35]
Cost of an MRI	199	Fixed	[35]
Cost of an EEG	97	Fixed	[35]
Cost of CSF testing	19	Fixed	[35]
Cost of an EITB test	98	Fixed	[35]
Cost of an ELISA	30	Fixed	[35]
Cost of a one-day stay in a hospital’s general ward	65	Fixed	[35]
Cost of a one-day stay in a hospital’s private ward	77	Fixed	[35]
Cost of surgery (ventriculoperitoneal shunt placement or cyst removal)	1,455	Fixed	[35]
Cost of a visit to a traditional healer to treat epilepsy	Min: 1 Mode: 2 Max: 8	Triangular	S2 Supporting Information
Cost of a visit to a traditional healer to treat severe chronic headaches	Min: 0.5 Mode: 2 Max: 8	Triangular	S2 Supporting Information
Minimum wage (per day)	4.3	Fixed	[39]
Median wage in urban area (per day)	15.8	Fixed	[38]
Median wage in rural area (per day)	7.9	Fixed	[see text]
Price of an adult pig	96	Fixed	[42]
Percent reduction in the price of a pig with cysticercosis	Min: 20 Max: 30	Uniform	[7]

Indirect costs included loss of working days due to visits to a healthcare provider, hospitalization or illness not requiring immediate medical attention, and loss of income due to reduced productivity. The 2015 median wage was applied to lost working days for those who were officially employed outside of the home in urban areas [38,39]. According to the 2016 Mexico National Household Income and Expenditure Survey, urban households had an average income more than double that of the rural households [40]. Therefore, for those people who lived in rural areas and were officially employed outside of the home, half the urban median wage was applied. The 2015 minimum wage was applied to lost working days for those who were not employed outside of the home in both urban and rural areas [39]. To capture the productivity losses of the unemployed population, excluding retirees, the minimum wage approach was used where time lost was estimated at an 8-hour workday. The proportion of the population that was not economically active in 2015 was obtained from Mexico's Instituto Nacional de Estadistica y Geografia [31]. It was assumed that losses for a child less than 15 years of age would be the same as for an adult since a parent would need to take time off work or would lose productivity while caring for the child.

Loss of future years of income and productivity, due to NCC-associated deaths, was calculated based on a 2012 study [17]. In 2012, NCC cases resulted in an estimated 7,062 years of life lost due to premature mortality in Mexico [17]. After adjusting for the increased population size in 2015, this value increased to 13,575 years of life lost. Three approaches were used to capture wage and productivity losses due to premature mortality. The first method used the minimum wage approach where time lost was estimated at an 8‐hour workday paid at Mexico's 2015 minimum wage of U.S.$4.30 per day [39]. The second method used the median wage approach where time lost was estimated at an 8‐hour workday paid at Mexico's 2015 median wage of U.S.$15.80 per day [38]. For the third method, time lost was estimated using the gross domestic product (GDP) per capita (U.S.$9,298) [41]. All three approaches calculated losses based on life expectancy in Mexico.

The price of an average finished live pig (weighing 150 lb) in Mexico in 2015 was obtained from the Food and Agriculture Organization of the United Nations [42]. The average reduction in the price of a cysticercosis-infected pigs, regardless of slaughter location, was estimated at 20–30% of market value based on information from the only identified study of its kind, which was conducted in Africa [7]. A 2015 exchange rate of 16.51 Mexican pesos for 1 U.S. dollar was used for all estimates [43].

### Analysis

Economic losses due to NCC-associated epilepsy and severe chronic headaches, with 95% credible regions (95% CRs), were estimated using @Risk (Palisade Corporation, Ithaca, NY, version 5.7). Latin Hypercube sampling was used for uncertain parameters. The model was run for 20,000 iterations to achieve convergence. Uncertain epidemiologic and economic parameters were modeled using normal, uniform, and triangular distributions. Regression sensitivity analysis was conducted in @Risk by varying the value of each parameter to estimate its correlation to the total cost estimate. The relative values of the regression coefficients indicate which parameters had the greatest impact on the total cost estimate.

### Ethics statement

This study received IRB approval from Texas A&M University (2006–0606 and 2014–0702), the Instituto Nacional de Neurologia y Neurocirugia (INNN), and the Hospital de Especialidades of the Instituto Mexicano del Seguro Social (HE-IMSS).

## Results

Nearly half a million people were estimated to suffer from NCC-associated epilepsy and severe chronic headaches in Mexico in 2015. Details on the number of cases according to their treatment patterns are shown in Table 5.

**Table 5:** Estimated number of NCC-associated epilepsy and NCC-associated severe chronic headaches cases in 2015 in Mexico along with their 95% Credible Regions (CR).

Final level of care	Hospitalized	Symptom	Number of NCC patients	95% CR
Primary care clinic	No	Epilepsy	111,944	56,559–191,264
		Severe chronic headaches	67,237	22,763–149,346
Secondary care clinic	Yes	Epilepsy	17,416	2,420–52,144
		Severe chronic headaches	2,991	407–9,477
	No	Epilepsy	25,394	4,079–65,171
		Severe chronic headaches	9,620	2,328–25,782
Tertiary care hospital	Yes	Epilepsy	7,946	1,363–20,067
		Severe chronic headaches	1,381	172–4,438
	No	Epilepsy	18,542	3,180–46,823
		Severe chronic headaches	711	89–2,386
Exclusively traditional healer	No	Epilepsy	45,803	1,614–126,183
		Severe chronic headaches	35,466	972–139,760
No treatment	No	Epilepsy	80,538	203–182,406
		Severe chronic headaches	74,467	3,588–240,933
*Total			499,456	254,943–857,791

* Note: Of this total, 49,748 (95% CR: 12,858–111,217) people are estimated to have received care from both a modern medical facility and a traditional healer.

### Monetary losses incurred by people with NCC-associated epilepsy and severe chronic headaches who received modern medical treatment

The total estimated losses incurred by people with NCC-associated epilepsy and severe chronic headaches who received treatment at a primary care clinic was U.S.$51,735,344 (95% CR: U.S.$21,905,869 –U.S.$98,121,733), with U.S.$288 (95% CR: U.S.$173 –U.S.$427) lost per patient of which 53% was due to indirect costs. Total estimated losses for people with NCC-associated epilepsy and severe chronic headaches who received treatment at a secondary care clinic was U.S.$41,909,153 (95% CR: U.S.$13,681,434 –U.S.$92,952,715), with U.S.$750 (95% CR: U.S.$515 –U.S.$1,074) lost per patient of which 38% was due to indirect costs. Total losses incurred by people with NCC-associated epilepsy and severe chronic headaches who received treatment at a tertiary care hospital was U.S.$37,572,440 (95% CR: U.S.$11,346,254 –U.S.$88,345,267), with U.S.$1,313 (95% CR: U.S.$476 –U.S.$2,157) lost per patient of which 30% was due to indirect costs. Tables 6 and 7 show the total direct and indirect losses and the cost per patient associated with individuals with NCC-associated epilepsy and severe chronic headaches who received modern medical treatment.

**Table 6:** Total direct costs and the cost per patient for people with NCC-associated epilepsy and NCC-associated severe chronic headaches who received modern medical treatment in 2015 along with their 95% CRs (in 2015 U.S.$).

Final level of care	Symptoms	Hospitalized	Total direct cost	Cost per patient
Primary care clinic	Epilepsy	No	18,423,641 (7,393,267–37,611,453)	164 (98–264)
	Severe chronic headaches	No	5,962,497 (921,662–18,355,313)	88 (22–193)
Secondary care clinic	Epilepsy	Yes	13,976,906 (1,942,616–42,072,286)	802 (696–969)
		No	8, 897,179 (1,328,422–24,740,045)	350 (244–517)
	Severe chronic headaches	Yes	1,444,825 (278,237–4,301,558)	521 (413–832)
		No	1,697,251 (390,044–4,698,337)	176 (131–234)
Tertiary care hospital	Epilepsy	Yes	13,430, 803 (2,303,428–33,916,390)	1,690 (1472–1,927)
		No	10, 190,428 (1,747,693–25,733,576)	550 (243–896)
	Severe chronic headaches	Yes	2,514,205 (313,237–8,081,744)	1,821 (1,693–1,949)
		No	324,153 (40,385–1,041,967)	456 (378–557)
Traditional healer cost for those who received treatment from both a traditional healer and a modern doctor	Epilepsy	No	222,037 (23,151–736,825)	6 (2–14)
	Severe chronic headaches	No	54,425 (3,054–215,889)	4 (1–9)

**Table 7:** Total indirect costs and the cost per patient for people with NCC-associated epilepsy and NCC-associated severe chronic headaches who received modern medical treatment in 2015 along with their 95% CRs (in 2015 U.S.$).

Final level of care	Symptoms	Hospitalized	Total cost	Cost per patient
Primary care clinic	Epilepsy	No	18,889,949 (3,303,171–45,450,819)	167 (34–328)
	Severe chronic headaches	No	8,459,260 (1,337,782–22,918,286)	126 (28–225)
Secondary care clinic	Epilepsy	Yes	8,737,000 (184,955–26,401,166)	499 (470–511)
		No	5,136,503 (802,240–13,337,565)	201 (190–206)
	Severe chronic headaches	Yes	916,619 (123,194–2,927,854)	306 (281–315)
		No	1,245,301 (296,480–3,366,155)	129 (118–132)
Tertiary care hospital	Epilepsy	Yes	5,368,738 (860,600–13,690,712)	670 (620–685)
		No	5,051,232 (809,704–12,881,047)	270 (250–276)
	Severe chronic headaches	Yes	569,379 (67,311–1,849,506)	408 (359–420)
		No	123,502 (14,600–401,169)	172 (151–177)

### Monetary losses and the cost per case associated with people with NCC-associated epilepsy and severe chronic headaches who did not receive modern medical treatment

The total monetary losses for people with NCC-associated epilepsy and NCC-associated severe chronic headaches who did not receive any treatment in Mexico in 2015 was U.S.$57,744,271 (95% CR: U.S.$14,615,051 –U.S.$143,750,099) and U.S.$25,674,058 (95% CR: U.S.$5,724,834 –U.S.$72,571,944), with U.S.$454 (95% CR: U.S.$154 –U.S.$913) and U.S.$237 (95% CR: U.S.$104 –U.S.$455) lost per patient, respectively. The total monetary losses for people with NCC-associated epilepsy and NCC-associated severe chronic headaches who exclusively received treatment from a traditional healer was U.S.$640,301 (95% CR: U.S.$74,556 –U.S.$1,891,517) and US$223,024 (95% CR: U.S.$16,260 –U.S.$851,633), with U.S.$14 (95% CR: U.S.$6 –U.S.$28) and U.S.$6 (95% CR: U.S.$2 –U.S.$14) lost per patient, respectively.

### Loss of future years of income and productivity due to NCC-associated deaths

Loss of future years of income and productivity due to NCC-associated deaths was estimated at U.S.$14.68 million, U.S.$54.26 million, and U.S.$126.22 million based on the minimum wage, median wage, and GDP per capita approaches, respectively.

### Pig losses

Monetary losses associated with porcine cysticercosis were estimated at U.S.$19,507,171 (95% CR U.S.$5,734,782 –U.S.$35,913,487) in 2015.

### Total economic losses

The total 2015 monetary losses associated with NCC-associated epilepsy and NCC-associated severe chronic headaches morbidity along with losses to the agriculture sector, was estimated to be U.S.$235,282,227 (95% CR U.S.$128,732,265 –U.S.$379,204,670), with U.S.$436 (95% CR: U.S.$296 –U.S.$604) lost per patient of which 60% was due to indirect costs. When loss of future years of income and productivity due to NCC-associated deaths based on the median wage approach was included, the estimate increased to U.S.$289,864,009 (95% CR U.S.$182,249,456 –U.S.$439,210,431).

### Sensitivity analysis

**Fig 2:**
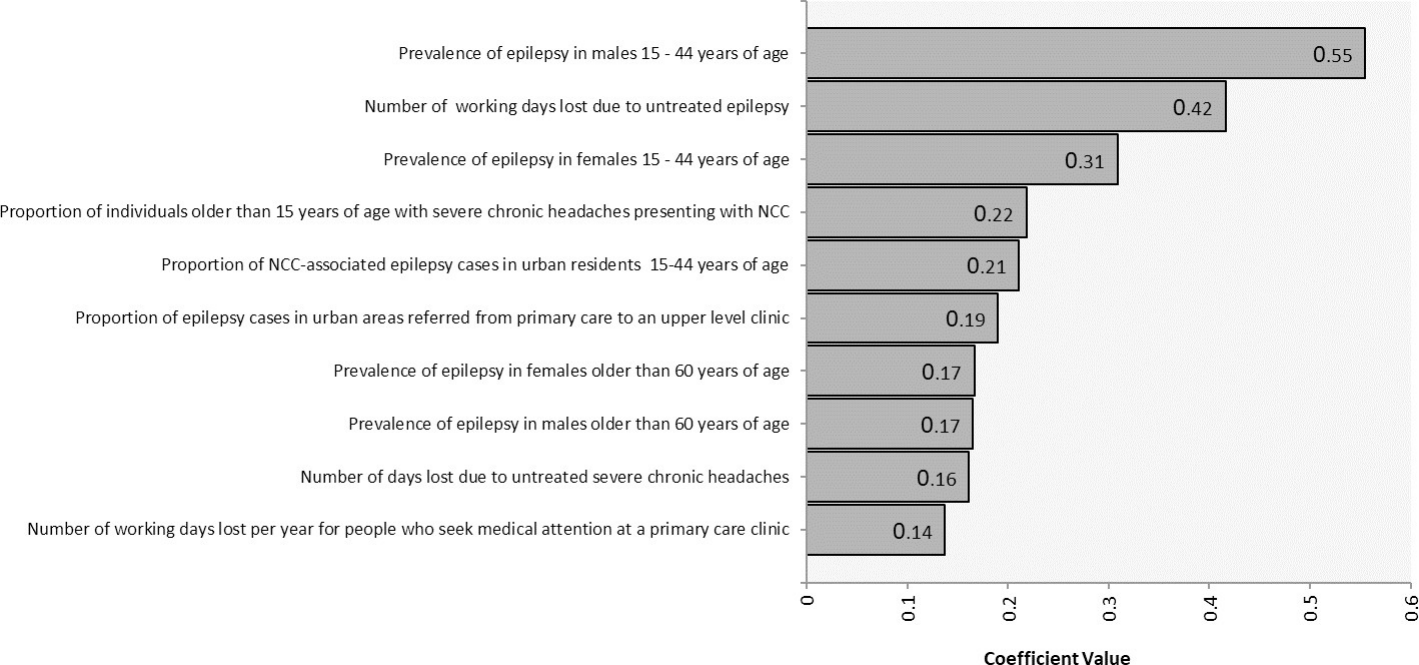
Sensitivity analysis for the estimated cost of cysticercosis in Mexico.

Fig 2 shows how uncertain parameters influenced the total monetary burden estimate. Prevalence of epilepsy in 15–44 year-old males and females and number of working days lost due to untreated epilepsy were the three parameters with the greatest effect on the total cost estimate.

## Discussion

This study is the first to estimate the monetary burden of cysticercosis incurred by people with NCC-associated epilepsy and severe chronic headaches, as well as pig-associated losses, in Mexico. Table 8 summarizes estimations of monetary losses due to *T*. *solium* cysticercosis in Mexico (the current study) and studies conducted in West Cameroon, the Eastern Cape Province of South Africa, and Tanzania [3,4,10].

**Table 8:** Comparison of disease burden estimates due to *T*. *solium* cystercosis in Mexico with other countries.

Estimate	Mexico (This study)	Eastern Cape Province, South Africa [3]	West Cameroon [4]	Tanzania [10]
**Study year**	2015	2004	2009	2012
**Country population**	125,235,587	7,088,000	5,065,382	44,928,923
**Estimated number of NCC-associated epilepsy cases**	307,583	34,662	50,326	47,804
**Overall monetary burden, including NCC-associated epilepsy morbidity losses and pig losses (in U.S. dollars)** **% due to porcine cysticercosis**	**1**86.2 million 10.5%	18.6–34.2 million** 14.6–26.9%	14.9 million* 4.7%	7.9 million 35.4%
**Average cost per NCC-associated epilepsy patient (U.S. dollars)**	542	632–844	240	106
**Average cost per capita (U.S. dollars)**	**1.**5	2.6–4.2	2.9	0.176

* based on a 2009 exchange rate of 1 U.S.$ = 0.69 Euro

** The range is due to the application of different calculation methods for wage and productivity losses (mean wage approach, generalist replacement costs, and opportunity costs).

The overall monetary burden reported in the current Mexican study was higher than what was reported in the South African, West Cameroon, and Tanzanian studies. However, these studies only accounted for the disease burden due to human NCC-associated epilepsy and pig losses and not for severe chronic headaches. When only NCC-associated epilepsy and pig losses are considered, the Mexico estimate falls to U.S.$**1**86.2 million, which is still considerably higher than the previous estimates. However, the cost per epilepsy patient (U.S.$542) is similar to the estimate produced for South Africa and higher than the estimates for Cameroon and Tanzania. This may be due to lower salaries and treatment costs in Cameroon and Tanzania. Indeed, compared to Tanzania and West Cameroon, the cost of a visit to the hospital, doctor or traditional healer was higher in Mexico. Also, patients in South Africa and Mexico may have better access to diagnostic tests, such as CT and MRI, and surgical interventions compared to patients in Tanzania and Cameroon.

In the current study, the median wage was used to value productivity losses of all economically active individuals and Mexico’s minimum wage was used to value productivity losses of all economically inactive individuals. The minimum wage approach was used since these individuals do contribute to society even though they are not formally employed outside of the home and make-up about forty percent of the population. In contrast, the South African study used three approaches (the mean wage approach, opportunistic cost approach, and the generalist replacement costs approach) to calculate productivity losses whereas the Cameroon and Tanzanian studies used the minimum and maximum salary and applied either a uniform or a gamma distribution. In the Mexican study, a large proportion (60%) of the total cost was related to indirect costs, which is in line with the conclusions of the South African, Cameroon, and Tanzanian studies.

NCC patients with epilepsy symptoms incurred larger annual monetary losses than those with severe chronic headaches. This may be due to the higher costs associated with epilepsy drugs compared to drugs used to treat severe chronic headaches. Other reasons might be the higher frequency of doctor visits and additional working days lost compared to patients with severe chronic headaches. In contrast, hospitalized NCC patients with severe chronic headaches incurred larger per-person monetary losses than those with epilepsy. This was due to the lower number of patients with NCC-associated epilepsy who had surgery compared to the number with severe chronic headaches who had surgery. The annual monetary losses per untreated NCC-associated epilepsy or untreated NCC-associated severe chronic headaches case were higher than the annual losses for their counterparts who received treatment at a primary care clinic in Mexico. This was due to a greater number of lost working days for those people not receiving any form of treatment.

Based on the regression sensitivity analysis, the most influential parameters were prevalence of epilepsy in 15–44 year-olds and the number of working days lost due to untreated epilepsy. The epilepsy prevalence estimates were based on a single study that may not fully reflect the regional variation in epilepsy cases. Numbers of days lost due to untreated epilepsy were based on questionnaire responses obtained from people who worked in the Ministry of Health in Michoacán, with the obtained values having quite large ranges. Studies on the impact of NCC on productivity are needed for both treated and untreated individuals to refine estimates of disease burden.

Our study has some limitations. The model most likely overestimated the costs associated with people manifesting both epilepsy and severe chronic headaches since the model assumes that costs associated with these two conditions were additive, which is most likely not the case. However, the total estimated cost was most likely underestimated since only the NCC-associated clinical manifestations of epilepsy and severe chronic headaches were included. Other neurological manifestations of NCC, such as stroke and dementia may also carry a significant burden, but were not included due to the absence of valid frequency data. Costs associated with family members who may accompany adults with NCC to clinics or hospitals were also not included due to the absence of reliable data. Moreover, this study relied on responses provided by physicians working in primary care clinics, neurologists working in secondary and tertiary care clinics, and employees at the Office of the Ministry of Health in Michoacán. Since these values come from a single endemic region, they may not be applicable to the entire country. The uncertainty placed around these parameters and the findings of the sensitivity analysis suggest that additional studies about healthcare-seeking behavior and treatment gaps are needed.

Burden of disease estimates, using the disability adjusted life year (DALY), not only consider losses due to morbidity, but also losses due to mortality. When monetized, these values appear to be substantial. However, it is difficult to ascertain the actual lost value to society for an individual, such as a rural manual laborer, whose position may be readily replaced by an able-bodied worker. It is also difficult to estimate the losses in revenue following retirement. Nonetheless, these estimates further support the fact that the cost of NCC in Mexico is still considerable.

Due to the absence of data evaluating how infection affects the cost of pigs that are not slaughtered in formal settings, it was assumed that there would be a reduction across all settings. This was also the assumption for the South African and Cameroon studies [6]. However, losses due to porcine cysticercosis in non-inspected facilities may be variable depending on whether or not there is suspicion of cysticercosis. If only losses in inspected pigs were assumed, pig-associated losses would decrease from U.S.$19,507,171 to U.S.$78,250.

It should be noted that this study only incorporated medical and agricultural costs. However, NCC also produces social impacts on an individual and community level that were not captured in the current study. For example, patients with epilepsy or severe chronic seizures are often stigmatized. Entire communities may also face stigmatization if they are known for producing infected pigs, thereby reducing the marketability of their animals. Therefore, the true societal burden is likely higher than that estimated by this study. On the other hand, it has been suggested that cysticercosis may be a diminishing problem in Mexico due to the implementation of control programs and improved living conditions [44]. Therefore, improvement in socioeconomic conditions may be resulting in decreasing numbers of infected humans and pigs, at least on a regional level.

This preliminarily estimate suggests that *T*. *solium* cysticercosis results in considerable monetary losses in Mexico even when compared to other diseases. For example, a study showed that the monetary burden of dengue in Mexico was U.S.$170 million in 2010. Although the estimated number of people affected by dengue was three times lower than the estimated number with cysticercosis, the cost was similar because surveillance and vector control accounted for 48.9% of the total economic burden of dengue [45]. In conclusion, this is a first study to estimate the monetary burden of cysticercosis in Mexico. The methodology developed here can be applied to estimate the monetary burden of cysticercosis in other regions in order to better prioritize disease control initiatives.

## Supporting information

S1 Supporting Information Epidemiologic parameters used to estimate the number of NCC cases with epilepsy and severe chronic headaches. (DOCX)

S2 Supporting Information Setting-specific questionnaire to obtain information on healthcare-seeking behavior and treatment gap parameters in Mexico. (DOCX)

S3 Supporting Information Parameters associated with the use of healthcare resources, including diagnostic testing and prescribed medications, by people with NCC-associated epilepsy and severe chronic headaches. (DOCX)
