# Supplementary material for: The monetary burden of cysticercosis in Mexico
Source: PLoS Negl Trop Dis. 2019 Jul 10;13(7):e0007501. doi: 10.1371/journal.pntd.0007501 (PMC6645581; doi:10.1371/journal.pntd.0007501)
Supplement: S1 Supporting Information — (DOCX) [file pntd.0007501.s001.docx]

**Supporting Information 1: Epidemiologic parameters used to estimate the number of NCC cases with epilepsy and severe chronic headaches**

The number of epilepsy cases was estimated by multiplying the age and rural/urban stratified population size from the 2015 census by the epilepsy prevalence estimates for Mexico [[1](#_ENREF_1)]. The number of NCC-associated epilepsy cases was obtained by multiplying the estimated numbers of people with epilepsy in rural and urban areas by the respective proportion of people with epilepsy with NCC lesions seen at CT-scan based on a meta-analysis of NCC-frequency data from Latin America [[2](#_ENREF_2)]. The results from this meta-analysis were also used to estimate the number of NCC-associated epilepsy cases receiving modern medical treatment in urban and rural areas. This was achieved by multiplying the numbers of NCC-associated epilepsy cases in rural and urban areas by the respective percentages seeking treatment [[2](#_ENREF_2)].

The proportion of NCC cases with severe chronic headaches was estimated using a multistep process. First, the total number of NCC cases presenting to a healthcare facility for any NCC-associated symptom (epilepsy, severe chronic headaches, focal deficits, stroke, dementia, etc.) was estimated. This was done by dividing the estimated number of NCC-associated epilepsy cases seeking treatment (see above) by the proportion of all symptomatic individuals with NCC who present to neurological clinics with epilepsy reported in a meta-analysis of clinical manifestations associated with NCC [[3](#_ENREF_3)]. Next, the number of people with NCC-associated severe chronic headaches presenting to healthcare facilities was obtained by multiplying the total number of NCC cases presenting to a healthcare facility for any NCC-associated symptom (stratified by urban/rural origin) by the proportion of NCC cases presenting with headaches at neurological clinics based on the same systematic review [[3](#_ENREF_3)]. Finally, the total number of people with NCC-associated severe chronic headaches, in urban and rural areas, was calculated by dividing the total number of NCC-associated severe chronic headaches cases seen in healthcare facilities by the proportion of NCC cases with severe chronic headaches who received treatment in a neurology clinic based on Carabin et al. 2011 [[3](#_ENREF_3)]. The proportion of people with symptomatic NCC that have epilepsy or severe chronic headaches was assumed to be the same regardless of whether they were seen at a primary, secondary, or tertiary care facility, due to the lack of data for individuals treated at different levels in Mexico. It should be noted that some individuals with NCC have both epilepsy and severe chronic headaches and these people contribute to the estimates for both NCC-associated epilepsy and NCC-associated severe chronic headaches.

**Table S1-A: Epidemiological parameters used to calculate the number of NCC-associated epilepsy and severe chronic headache cases**

| **Parameter** | **Value or range of values** | **Distribution** | **Reference** |
| --- | --- | --- | --- |
| 2015 Population of Mexico ('000) |  |  |  |
| Total | 125,235,587 | Fixed | [[4](#_ENREF_4)] |
| Urban areas | 99,244,722 | Fixed | [[4](#_ENREF_4)] |
| Rural areas | 25,990,865 | Fixed | [[4](#_ENREF_4)] |
| Prevalence of epilepsy in 0-14-year-old males in Mexico (per 1,000) | Min:1.4  Max:12.5 | Uniform  (1.4 - 12.5) | [[1](#_ENREF_1)] |
| Prevalence of epilepsy in 0-14-year-old females in Mexico (per 1,000) | Min: 0.8  Max: 10.0 | Uniform  (0.8 - 10.0) | [[1](#_ENREF_1)] |
| Prevalence of epilepsy in 15-44-year-old males in Mexico (per 1,000) | Min: 1.4  Max: 17.2 | Uniform  (1.4 - 17.2) | [[1](#_ENREF_1)] |
| Prevalence of epilepsy in 15-44-year-old females in Mexico (per 1,000) | Min: 1.4  Max: 11.7 | Uniform  (1.4 - 11.7) | [[1](#_ENREF_1)] |
| Prevalence of epilepsy in 45-59-year-old males and females in Mexico (per 1,000) | Min: 0.1  Max: 13.2 | Uniform  (0.1 - 13.2) | [[1](#_ENREF_1)] |
| Prevalence of epilepsy in males and females older than 60 years of age in Mexico (per 1,000) | Min: 0.3  Max: 30.8 | Uniform  (0.3 - 30.8) | [[1](#_ENREF_1)] |
| Proportion of epilepsy cases associated with NCC in urban area of Mexico | Min: 0.21  Max: 0.37 | Uniform  (0.21 - 0.37) | [[2](#_ENREF_2)] |
| Proportion of epilepsy cases associated with NCC in rural areas of Mexico | Min: 0.26  Max: 0.49 | Uniform  (0.26 - 0.49) | [[2](#_ENREF_2)] |
| Proportion of NCC patients 0-14 years of age with epilepsy | Min: 0.70  Max: 0.86 | Uniform  (0.70 - 0.86) | [[3](#_ENREF_3)] |
| Proportion of NCC cases older than 15 years of age with epilepsy | Min: 0.52  Max: 0.74 | Uniform  (0.52 - 0.74) | [[3](#_ENREF_3)] |
| Proportion of people with epilepsy not receiving modern medical treatment in urban areas | Min: 0.10  Max: 0.46 | Uniform  (0.10 - 0.46) | [[2](#_ENREF_2)] |
| Proportion of people with epilepsy not receiving modern medical treatment in rural areas | Min: 0.67  Max: 0.87 | Uniform  (0.67 - 0.87) | [[2](#_ENREF_2)] |
| Proportion of people with severe chronic headaches not receiving modern medical treatment in urban areas | Min: 0.20  Max: 0.56 |  | [see text] |
| Proportion of people with severe chronic headaches not receiving modern medical treatment in rural areas | Min: 0.67  Max: 0.87 |  | [see text] |
| Proportion of people 0-14 years of age with severe chronic headaches presenting with NCC | Min: 0.21  Max: 0.35 | Uniform  (0.21 - 0.35) | [[3](#_ENREF_3)] |
| Proportion of individuals older than 15 years of age with severe chronic headaches presenting with NCC | Min: 0.11  Max: 0.45 | Uniform  (0.11 - 0.45) | [[3](#_ENREF_3)] |

**Table S1-B: Estimated numbers of people with NCC-associated epilepsy and severe chronic headaches in Mexico**

| **NCC-associated manifestation** | **Area** | **Number** | **95% CR** |
| --- | --- | --- | --- |
| Epilepsy | Urban | 201,835 | 116,524 - 298,915 |
|  | Rural | 77,652 | 47,665 - 111,448 |
| Severe chronic headaches | Urban | 100,008 | 37,555 - 222,178 |
|  | Rural | 72,867 | 16,823 - 227,429 |

**References**

1. Quet F, Preux PM, Huerta M, Ramirez R, Abad T, et al. (2011) Determining the burden of neurological disorders in populations living in tropical areas: who would be questioned? Lessons from a Mexican rural community. Neuroepidemiology 36: 194-203.

2. Bruno E, Bartoloni A, Zammarchi L, Strohmeyer M, Bartalesi F, et al. (2013) Epilepsy and neurocysticercosis in Latin America: a systematic review and meta-analysis. PLoS Negl Trop Dis 7: e2480.

3. Carabin H, Ndimubanzi PC, Budke CM, Nguyen H, Qian Y, et al. (2011) Clinical manifestations associated with neurocysticercosis: a systematic review. PLoS Negl Trop Dis 5: e1152.

4. UN Data (2010) UN Data2010 Available: http://data.un.org/Data.aspx?d=POP&f=tableCode:22 Accessed 08 December 2015. Available: http://data.un.org/Data.aspx?d=POP&f=tableCode:22 Accessed 08 December 2015.
