# Supplementary material for: The monetary burden of cysticercosis in Mexico
Source: PLoS Negl Trop Dis. 2019 Jul 10;13(7):e0007501. doi: 10.1371/journal.pntd.0007501 (PMC6645581; doi:10.1371/journal.pntd.0007501)
Supplement: S2 Supporting Information — (DOCX) [file pntd.0007501.s002.docx]

**Supporting Information 2: Setting-specific questionnaire to obtain information on healthcare-seeking behavior and treatment gap parameters in Mexico**

Due to a lack of published literature on several healthcare seeking behavior and treatment gap parameters, a setting-specific questionnaire was developed in Spanish to obtain information not found in the published literature. Most of the questions were open-ended. Michoacán is considered an endemic area for cysticercosis in Mexico [[1](#_ENREF_1)] [[2](#_ENREF_2)]. Therefore, individuals working at each of the following facilities located in the state of Michoacán were selected to complete the questionnaire in 2014: the Ministry of Health, one primary care clinic, and one secondary care clinic. More than 10 responses were received from each setting. However not everyone responded to all questions. The table below shows the number of responses in addition to the minimum, mode, and maximum values obtained for each question.

**Table S2-A: Data obtained from individuals working at the Michoacán Ministry of Health**

|  | **Number of responses** | **Minimum** | **Mode** | **Maximum** |
| --- | --- | --- | --- | --- |
| In rural areas, what proportion of epilepsy patients do you believe consult a traditional healer before consulting a physician? | 13 | 0.01 | 0.05 | 1 |
| What proportion of epilepsy patients do you think sees a traditional healer without ever consulting a modern doctor? | 19 | 0 | 0 | 0.7 |
| In rural areas, what proportion of severe chronic headaches patients do you believe consult a traditional healer before consulting a physician? | 15 | 0 | 0 | 0.95 |
| What proportion of patients with severe chronic headaches do you think sees a traditional healer without ever consulting a modern doctor? | 18 | 0 | 0 | 0.8 |
| How much does a traditional healer typically charge to treat epilepsy? | 12 | US$ 1 | US$2 | US$8 |
| How much does a traditional healer typically charge to treat severe chronic headaches? | 11 | US$0.5 | US$2 | US$8 |
| How many days of work (or school) do you think a person with untreated severe chronic headaches misses every month? | 14 | 1 | 2 | 10 |
| How many days of work (or school) do you think a person with untreated epilepsy misses every month? | 13 | 1 | 1 | 5 |

**Table S2-B: Data obtained from physicians at a primary care clinic in Michoacán**

|  | **Number of responses** | **Minimum** | **Mode** | **Maximum** | **Comments** |
| --- | --- | --- | --- | --- | --- |
| What proportion of your patients with epilepsy is referred directly to a tertiary care hospital? | 12 | 0 | 0 | 0.4 |  |
| What proportion of patients with epilepsy that seek treatment at your clinic is hospitalized at your clinic? | 20 | - | - | - | No hospital beds in clinic |
| What are the principal drugs provided to/used by patients with epilepsy who are seen at your clinic? If possible, please include dosages. | 20 | - | - | - | Phenytoin  Carbamazepine  Valproic acid |
| How many times per year do you think patients with epilepsy consult medical doctors at a primary care clinic? | 18 | 1 | 2 | 12 |  |
| What proportion of patients with severe chronic headaches seeks medical attention at a primary care clinic? | 16 | 1 | 1 | 12 |  |
| What proportion of your patients with severe chronic headaches is referred directly to a tertiary care hospital? | 14 | 0 | 0 | 0.1 |  |
| What proportion of patients with severe chronic headaches that seek treatment at your clinic is hospitalized at your clinic? | 20 | - | - | - | No hospital beds in clinic |
| What are the principal drugs provided to/used by patients with severe chronic headaches who are seen at your clinic? If possible, please include dosages. | 18 | - | - | - | Ketorolac tromethamine  Acetaminophen |
| How many times per year do you think patients with severe chronic headaches consult a medical doctor at a primary care clinic? | 15 | 1 | 1 | 12 |  |
| What tests are available at your clinic for the diagnosis of NCC? | None/X-ray |  |  |  |  |
| How many days of work (or school) do you think a person treated at your clinic for severe chronic headaches misses every month? | 15 | 0 | 1 | 3 |  |
| How many days of work (or school) do you think a person treated at your clinic for severe headache misses every month? | 14 | 0 | 1 | 2 |  |

**Table S2-C: Data obtained from physicians at a secondary care hospital in Michoacán**

|  | **Number of responses** | **Minimum** | **Mode** | **Maximum** | **Comments** |
| --- | --- | --- | --- | --- | --- |
| What proportion of your epilepsy patients do you refer to a tertiary care hospital? | 10 | 0 | 0 | 0.5 |  |
| What proportion of your patients with epilepsy is hospitalized at your facility? | 10 | 0.02 | 0.2 | 1 |  |
| What are the principal drugs provided to/used by your epilepsy patients? Please provide dosages if available. | 11 | - | - | - | Phenytoin  Carbamazepine  Valproic acid |
| What are the principal drugs prescribed for your patients with NCC? | 9 | - | - | - | Albendazole  Praziquantel |
| How many times per year do you think patients with epilepsy consult a medical doctor at a secondary care clinic? | 9 | 2 | 3 | 20 |  |
| What proportion of your patients with severe chronic headaches do you refer to a tertiary care hospital? | 10 | 0 | 0 | 0.25 |  |
| What proportion of your patients with severe chronic headaches is hospitalized at your facility? | 8 | 0.01 | 0.2 | 0.5 |  |
| What are the principal drugs provided to/used by your patients with severe chronic headaches? Please provide dosages if available. | 10 |  |  |  | Ketorolac tromethamine  Acetaminophen |
| How many times per year do you think patients with severe chronic headaches consult a medical doctor at a secondary care clinic? | 8 | 1 | 3 | 8 |  |
| What tests are available at your clinic for the diagnosis of? | 10 |  |  |  | CT, MRI and X-ray |
| How many days of work (or school) do you think a person treated at your hospital for severe chronic headaches misses every month? | 2 | 2 |  | 7 | Not enough data to calculate mode |
| How many days of work (or school) do you think a person treated at your hospital for epilepsy misses every month? | 2 | 1 |  | 10 | Not enough data to calculate mode |

**References**

1. Sarti Gutierrez E, Schantz PM, Aguilera J, Lopez A (1992) Epidemiologic observations on porcine cysticercosis in a rural community of Michoacan State, Mexico. Vet Parasitol 41: 195-201.

2. Sarti E, Schantz PM, Plancarte A, Wilson M, Gutierrez OI, et al. (1994) Epidemiological investigation of Taenia solium taeniasis and cysticercosis in a rural village of Michoacan state, Mexico. Trans R Soc Trop Med Hyg 88: 49-52.
