# Supplementary material for: The monetary burden of cysticercosis in Mexico
Source: PLoS Negl Trop Dis. 2019 Jul 10;13(7):e0007501. doi: 10.1371/journal.pntd.0007501 (PMC6645581; doi:10.1371/journal.pntd.0007501)
Supplement: S3 Supporting Information — (DOCX) [file pntd.0007501.s003.docx]

**Supporting Information 3: Parameters associated with the use of healthcare resources, including diagnostic testing and prescribed medications, by people with NCC-associated epilepsy and severe chronic headaches**

**Table S3-A. Parameters associated with the use of healthcare resources (per year) in people with NCC-associated epilepsy or severe chronic headaches in Mexico**

| **Parameter** | **Symptom** | **Value** | **Distribution** | **Reference** |
| --- | --- | --- | --- | --- |
| Number of visits to a traditional healer by a patient who also receives treatment from a modern doctor | Epilepsy | Min: 2  Max: 3 | Uniform | [see text] |
|  | Severe chronic headaches | Min: 1  Max: 2 | Uniform | [see text] |
| Number of visits to a traditional healer by a patient who exclusively seeks treatment from a traditional healer | Epilepsy | Min: 4  Max: 6 | Uniform | [see text] |
|  | Severe chronic headaches | Min: 2  Max: 3 | Uniform | [see text] |
| Number of visits to a physician by a patient who seeks treatment at a primary care clinic | Epilepsy | Min: 1  Mode: 2  Max: 12 | Triangular | Table S2-B |
|  | Severe chronic headaches | Min: 1  Mode: 1  Max: 12 | Triangular | Table S2-B |
| Number of visits to a neurologist by a patient who seeks treatment at a secondary care clinic | Epilepsy | Min: 2  Mode: 3  Max: 20 | Triangular | Table S2-B |
|  | Severe chronic headaches | Min: 1  Mode: 3  Max: 8 | Triangular | Table S2-B |
| Proportion of people treated at a tertiary care facility that receive a surgical intervention per year | Epilepsy | 0.25 | Fixed | [[1](#_ENREF_1)] |
|  | Severe chronic headaches | 0.57 | Fixed | [[1](#_ENREF_1)] |
| Proportion of people receiving treatment at a secondary care clinic who are hospitalized | Epilepsy | Min: 0.02  Mode: 0.2  Max: 1 | Triangular | Table S2-C |
|  | Severe chronic headaches | Min: 0.01  Mode: 0.2  Max: 0.5 | Triangular | Table S2-C |
| Length of a hospital stay (in days) for people who are hospitalized at a secondary care clinic | Epilepsy | 7 | Fixed | [see text] |
|  | Severe chronic headaches | 4 | Fixed | [see text] |
| Length of a hospital stay (in days) for people who are hospitalized at a tertiary care facility | Epilepsy | 10.96 | Fixed | [[1](#_ENREF_1)] |
|  | Severe chronic headaches | 7.56 | Fixed | [[1](#_ENREF_1)] |

**Table S3-B: Parameters associated with the use of diagnostic tests and prescription medications in people with NCC-associated epilepsy or severe chronic headaches in Mexico**

| **Parameter** | **Value** | **Distribution** | **Reference** |
| --- | --- | --- | --- |
| Proportion of people with epilepsy who seek medical attention at a primary, secondary or tertiary care clinic and are prescribed the anti-epileptic drug phenytoin | 0.95 | Fixed | [[1](#_ENREF_1)] |
| Proportion of people with epilepsy who seek medical attention at a primary, secondary or tertiary care clinic and are prescribed the anti-epileptic drug carbamazepine | 0.33 | Fixed | [[1](#_ENREF_1)] |
| Proportion of people with epilepsy who seek medical attention at a primary, secondary or tertiary care clinic and are prescribed the anti-epileptic drug valproic acid | 0.20 | Fixed | [[1](#_ENREF_1)] |
| Proportion of people with severe chronic headaches who seek medical attention at a primary, secondary or tertiary care clinic and are prescribed the anti-inflammatory drug ketorolac tromethamine | 0.37 | Fixed | [[1](#_ENREF_1)] |
| Proportion of people with severe chronic headaches who seek medical attention at a primary, secondary or tertiary care clinic and are prescribed the antipyretic drug acetaminophen | 0.26 | Fixed | [[1](#_ENREF_1)] |
| Proportion of people who are diagnosed with NCC and prescribed the anthelmintic drug albendazole at a secondary or tertiary care clinic | 0.36 | Fixed | [[1](#_ENREF_1)] |
| Proportion of people who are diagnosed with NCC and prescribed the anthelmintic drug praziquantel at a secondary or tertiary care clinic | 0.02 | Fixed | [[1](#_ENREF_1)] |
| Proportion of people who are diagnosed with NCC and receive a CT scan and/or MRI at a secondary care clinic | 0.18 | Fixed | Table S2-C |
| Proportion of people who are diagnosed with NCC-associated epilepsy and receive a CT scan at a tertiary care clinic | 0.42 | Fixed | [[1](#_ENREF_1)] |
| Proportion of people who are diagnosed with NCC-associated severe chronic headaches and receive a CT scan at a tertiary care clinic | 0.5 | Fixed | [[1](#_ENREF_1)] |
| Proportion of people who are diagnosed with NCC-associated epilepsy and receive an MRI at a tertiary care clinic | 0.77 | Fixed | [[1](#_ENREF_1)] |
| Proportion of people who are diagnosed with NCC-associated severe chronic headaches and receive an MRI at a tertiary care clinic | 0.76 | Fixed | [[1](#_ENREF_1)] |
| Proportion of people who are diagnosed with NCC-associated epilepsy and receive an EEG at a secondary or tertiary care clinic | 0.36 | Fixed | [[1](#_ENREF_1)] |
| Proportion of people who are diagnosed with NCC-associated severe chronic headaches and receive an EEG at a secondary or tertiary care clinic | 0.09 | Fixed | [[1](#_ENREF_1)] |
| Proportion of people who are diagnosed with NCC-associated epilepsy and receive EITB testing at a tertiary care clinic | 0.05 | Fixed | [[1](#_ENREF_1)] |
| Proportion of people who are diagnosed with NCC-associated severe chronic headaches and receive EITB testing at a tertiary care clinic | 0.007 | Fixed | [[1](#_ENREF_1)] |
| Proportion of people who are diagnosed with NCC-associated epilepsy and receive CSF testing at a secondary or tertiary care clinic | 0.33 | Fixed | [[1](#_ENREF_1)] |
| Proportion of people who are diagnosed with NCC-associated severe chronic headaches and receive CSF testing at a secondary or tertiary care clinic | 0.45 | Fixed | [[1](#_ENREF_1)] |
| Proportion of people who are diagnosed with NCC-associated epilepsy and receive ELISA testing at a tertiary care clinic | 0.33 | Fixed | [[1](#_ENREF_1)] |
| Proportion of people who are diagnosed with NCC-associated severe chronic headaches and receive ELISA testing at a tertiary care clinic | 0.42 | Fixed | [[1](#_ENREF_1)] |
| Proportion of people who are diagnosed with NCC-associated epilepsy and receive surgery at a tertiary care clinic | 0.25 | Fixed | [[1](#_ENREF_1)] |
| Proportion of people who are diagnosed with NCC-associated severe chronic headaches and receive surgery at a tertiary care clinic | 0.57 | Fixed | [[1](#_ENREF_1)] |

**Reference:**

1. Bhattarai R, Carabin H, Proano JV, Flores-Rivera J, Corona T, et al. (2015) Cost of neurocysticercosis patients treated in two referral hospitals in Mexico City, Mexico. Trop Med Int Health 20: 1108-1119.

1. B
